# Topographically and Chemically Enhanced Textile Polycaprolactone Scaffolds for Tendon and Ligament Tissue Engineering

**DOI:** 10.3390/polym16040488

**Published:** 2024-02-09

**Authors:** Benedict Bauer, Caroline Emonts, Johannes Pitts, Eva Miriam Buhl, Jörg Eschweiler, Robert Hänsch, Marcel Betsch, Thomas Gries, Henning Menzel

**Affiliations:** 1 Institut für Textiltechnik, RWTH Aachen University, Otto-Blumenthal-Straße 1, 52074 Aachen, Germany; caroline.emonts@ita.rwth-aachen.de (C.E.);; 2 Institute for Technical Chemistry, Braunschweig University of Technology, Hagenring 30, 38106 Braunschweig, Germany; 3 Institute of Pathology, Electron Microscopy Facility, RWTH University Hospital Aachen, Pauwelsstraße 30, 52074 Aachen, Germany; 4 Department of Trauma and Reconstructive Surgery, BG Hospital Bergmannstrost, Merseburgerstr. 165, 06112 Halle (Saale), Germany; joerg.eschweiler@bergmannstrost.de; 5 Department of Trauma and Reconstructive Surgery, University Hospital Halle, Ernst-Grube-Str. 40, 06120 Halle (Saale), Germany; 6 Institute of Plant Biology, Braunschweig University of Technology, Humboldtstraße 1, 38106 Braunschweig, Germany; 7 Department of Orthopaedics and Trauma Surgery, University Hospital Erlangen, Krankenhausstr. 12, 91054 Erlangen, Germany

**Keywords:** tendon, ligament, tissue engineering, degradation, PCL, surface modification, braiding, macroporous scaffold, Chitosan-graft-PCL, TGF release

## Abstract

The use of tissue engineering to address the shortcomings of current procedures for tendons and ligaments is promising, but it requires a suitable scaffold that meets various mechanical, degradation-related, scalability-related, and biological requirements. Macroporous textile scaffolds made from appropriate fiber material have the potential to fulfill the first three requirements. This study aimed to investigate the biocompatibility, sterilizability, and functionalizability of a multilayer braided scaffold. These macroporous scaffolds with dimensions similar to those of the human anterior cruciate ligament consist of fibers with appropriate tensile strength and degradation behavior melt-spun from Polycaprolactone (PCL). Two different cross-sectional geometries resulting in significantly different specific surface areas and morphologies were used at the fiber level, and a Chitosan-graft-PCL (CS-g-PCL) surface modification was applied to the melt-spun substrates for the first time. All scaffolds elicited a positive cell response, and the CS-g-PCL modification provided a platform for incorporating functionalization agents such as drug delivery systems for growth factors, which were successfully released in therapeutically effective quantities. The fiber geometry was found to be a variable that could be manipulated to control the amount released. Therefore, scaled, surface-modified textile scaffolds are a versatile technology that can successfully address the complex requirements of tissue engineering for ligaments and tendons, as well as other structures.

## 1. Introduction

The concept of tendon and ligament tissue engineering is a promising alternative to the use of autografts and permanent, synthetic grafts [1,2,3,4]. However, the requirements on the corresponding temporary support structure are manifold [2,5,6]. These include purely mechanical aspects, such as a sufficient breaking load. At the same time, the force–elongation behavior should match the native tissue as closely as possible while avoiding significant creep, slackening, or stress-shielding [4,7,8]. In addition, these mechanical properties must be maintained over a longer degradation period due to the slow healing rate of ligaments and tendons. For the anterior cruciate ligament (ACL), a strength retention of approx. 50% after 6 months is therefore recommended [9,10]. Besides the mechanical and degradation-specific aspects, morphological and biological aspects play a major role [11]. All of these requirements have not yet been satisfactorily met by any single material at once [6,12,13,14,15]. In principle, natural or synthetic polymers can be used. Natural polymers are frequently characterized as biologically superior to synthetic polymers [16]. However, deficits in the batch dependency of the base material, generally inferior mechanical properties, and processability compared to synthetic polymers limit the range of applications [4,7].

Among the synthetic polymers, polylactic acid (PLA), polyglycolic acid (PGA), polycaprolactone (PCL), and their copolymers have been used primarily to date. The advantages are unlimited availability, consistent quality, better controllable degradation, and excellent processability into (at least initially) mechanically highly functional structures [17,18,19]. However, excessive strength loss during degradation was reported [4,20,21]. In addition, the acidic degradation products of PLA and PGA have been associated with an increased inflammatory reaction [7,22,23]. This can be particularly detrimental in bradytrophic tissue [7]. Its biocompatibility, high initial strength and elasticity, and slow degradation rate, which results in no or only mild inflammatory reactions and retention of strength and elasticity during degradation, make PCL a promising material for tissue with low healing capacity and high mechanical demands [2,4,5,24]. In contrast to other forms, PCL has scarcely been investigated as melt-spun fibers [25]. As shown in earlier works, highly oriented melt-spun PCL fibers offer superb mechanical properties, sufficient strength retention during degradation, and processability to human ACL-sized scaffolds without exceeding the stiffness of the native tissue [26]. However, biological aspects have not been addressed yet. A promising approach is to combine high-strength melt-spun fibers with surface modifications that can offer biological cues. Leroux et al. used poly (sodium styrene sulfonate) (pNaSS) functionalization to improve the biological properties of PCL fibers [27,28,29]. Surface modification via the application of agents like collagen, chitosan, and gelatin also offers the possibility for increased biological performance and enables the introduction of agents such as growth factors to the desired scaffolds. Here, chitosan stands out regarding its comparably low price combined with several beneficial qualities, like accelerated wound healing and antibacterial properties [30,31]. Most of the other agents cannot provide these qualities combined. The attachment of chitosan groups to the PCL surface can be achieved via the comparably simple process of co-crystallization of the Chitosan-graft-PCL copolymer to the scaffold surface [32]. The high potential of Chitosan-graft-PCL surface modification was shown on electro-spun PCL fiber mats used as a rotator cuff scaffold, strongly increasing hydrophilicity, improving cell attachment, and allowing for further functionalization with a drug release system [32,33], which in turn is capable of releasing growth factors like BMP-2 and TGF-β_3_. The latter is known to induce chondrogenic differentiation in human mesenchymal stromal cells [34]. Until now, however, this surface modification has not been applied to yarn-based, high-strength PCL scaffolds.

Another potential pathway to promote cell growth is modifying fiber topography. By using non-circular fibers, the resulting grooves along the fiber axis can function as growth guidance for cells. Ramakrishna et al. have investigated the effect of deeply grooved PLA fibers, which were incorporated in a braided scaffold, on the proliferation of TGF-β-type-2 receptor-expressing progenitor cells. Besides a slight improvement in the biological properties, they observed a certain alignment of the cells within the grooves [35]. Park et al. compared three different cross-sections with increasing aspect ratios (circular, triangular, and cruciform) of extruded PCL strands in terms of tensile properties, degradation behavior, and cell proliferation. Compared with the circular strands, an increase in MG-63 (human osteosarcoma) cell proliferation of 112% and 50% after 7 days was observed for the cruciform and the triangular cross-sections, respectively [36]. However, due to the nature of the used extrusion device, only a little macromolecular orientation was added to the PCL strands, resulting in a very low tensile stress of approximately 14 MPa. To the best of our knowledge, melt-spun, non-circular PCL fibers have not yet been investigated in the context of tendon and ligament tissue engineering.

In this study, we investigate two paths to promote the biological performance of our PCL scaffolds by (1) applying a Chitosan-graft-PCL surface modification and/or (2) using non-circular fibers to induce a certain growth guidance. The potential of the increased relative surface area of non-circular multifilaments compared to circular monofilaments is further investigated in release tests with TGF-β_3_. This study is designed to address application-related aspects. Therefore, the macroporous, multilayer-braided scaffolds are fabricated to match the human ACL. Furthermore, a potential effect of the sterilization method on the mechanical and thermal properties is considered.

## 2. Materials and Methods

This section is divided into fabrication methods, cell culture, and analytics.

### 2.1. Fabrication of the Surface-Modified PCL Scaffold

#### 2.1.1. Preparation of PCL Fibers

Poly(ε-caprolactone) (PCL, M_n_ = 80,000 g/mol according to the supplier) was purchased from Sigma-Aldrich (St. Louis, MO, USA) in the form of polymer pellets. The spinning process was performed following our earlier work but with a research-grade polymer [26]. Monofilaments, as well as multifilaments (10 filaments per yarn), were fabricated using the melt spinning process (Fourné Polymertechnik GmbH, Alfter-Impekoven, Germany) at an extrusion temperature T_E_ = 222 °C. For monofilaments, a water bath (T_w_ = 19 °C ± 2 °C) was used for quenching. Multifilaments were cooled with a blow chamber, after which spin finish (Limanol LB25, Schill+Seilacher GmbH, Böblingen, Germany) in a concentration of 10 vol% in deionized water was applied. While the monofilaments were produced with a standard round die cross-section (Ø 0.5 mm, 2 L/d), a snowflake-shaped capillary geometry was chosen for the production of the multifilaments.

#### 2.1.2. Scaffold Fabrication

The braided scaffolds were produced using a circular braiding machine with fine wire carriers (type: HS80/48 from Körting Nachf. Wilhelm Steeger GmbH & Co. KG, Wuppertal, Germany). Each layer contains 48 filaments arranged in a 2:2_1 braiding pattern. A filament tension of 0.57 N was applied. The multilayer braids were made by overbraiding the previous layer to form two or four layers. The total filament count of the two-layer braids is n_F_ = 96 and n_F_ = 192 for the four-layer braids. All braids were made from round monofilament (ROMO) and snowflake multifilament (SFMU) fibers.

#### 2.1.3. Surface Modification and Nanoparticle Loading

Before modification, all scaffolds ((1 × 1.5 × 0.5) cm^3^) were washed to remove potential residues applied to their surface during production (e.g., spin finish). For this purpose, the fibers were placed into 45 mL centrifuge tubes, and 35 mL of PBS buffer was added. Each tube contained a maximum of 5 samples. The tubes were put vertically on a laboratory shaker running at 250 rpm for 15 min at room temperature (RT). Then, PBS was replaced with EtOH (aq, 70%) and washed for 1 h accordingly. This was repeated two more times, and, subsequently, the scaffolds were placed under a fume hood to dry out EtOH for 1 h. Finally, the scaffolds were washed three times with Milli-Q^®^ (Merck, Darmstadt, Germany) for 10 min each and dried overnight under a fume hood.

CS-g-PCL_56_ was synthesized in an improved and upscaled procedure based on de Cassan et al. [32] and Jing et al. [37] with the ratio x in CS-g-PCL_x_ calculated via the integral ratio in ^1^H-NMR according to Jing et al. The graft polymer (0.5 wt-%) was dissolved in acetic acid (aq, 77 vol-%) at 45 °C for 1 h. The solution (20 mL per 10 scaffolds) was transferred into a petri dish (Ø = 50 mm) and let to cool down to RT. The scaffolds were dipped in the solution for 2 min. Air trapped inside the denser scaffolds was removed by gentle shaking during the first 10 s of the procedure. Afterward, the scaffolds were removed from the solution, put into petri dishes, and placed into a vacuum oven at RT for 48 h. Finally, the scaffolds were washed with Milli-Q^®^ as performed before the modification process but this time in three intervals of 8 h each. Subsequent drying for 48 h under a fume hood finalized the process.

For modification with alginate, the CS-g-PCL_56_ scaffolds were dipped in an alginate solution (Milli-Q^®^, 5 mg/mL) for 2 min, removed, and rinsed with Milli-Q^®^. ε-Caprolactone was grafted onto a backbone of chitosan via a cationic ring-opening polymerization. Therefore, purified dry chitosan (350 mg, 2.09 mmol, 83% DDA, Mn = 190,000–310,000, Sigma-Aldrich) was dissolved in MeSO_3_H (2 mL) in a 250 mL dried Schlenk flask under a nitrogen atmosphere. Chitosan was dissolved after 45 min at 50 °C. In the next step, a ε-caprolactone monomer (15.9 mL, 168,46 mmol, 72 eq.) was added to the solution, and the mixture was stirred for 5 h at 50 °C. Finally, the nitrogen flow was stopped, and an aqueous quench solution containing 0.2 M KH_2_PO_4_ (43.75 mL), 10 M NaOH (7 mL), and 100 g of ice was added. The precipitated crude CS-g-PCL was collected by centrifugation and vacuum-dried for 48 h at RT. The dried CS-g-PCL was dissolved in N,N-dimethylformamide and reprecipitated in ice. It was then (again) collected by centrifugation and vacuum-dried at RT for 48 h. To carry this out, the scaffolds were immersed in Milli-Q^®^ (200 mL each, 10 scaffolds and beaker) and gently stirred for 30 min at RT. After removal from the beakers, the scaffolds were put into petri dishes and dried for 48 h under a fume hood at RT. Alginate fluorescein amine modification was performed similarly but with the alginate fluorescein amine solution (Milli-Q^®^, 5 mg/mL) being filtered through a membrane filter by WHATMAN™ (0.20 μm, polyamide, Merck, Darmstadt, Germany). Alginate fluorescein amine was provided by De Cassan et al. [32], who obtained it via a method described by Strand et al. using lyophilized sodium alginate (Sigma-Aldrich) [38]. In addition, washing with Milli-Q^®^ was performed until the supernatant no longer emitted fluorescence light in near darkness under a handheld UV lamp (366 nm). With Milli-Q^®^ being exchanged thrice a day, this took 5 d on a laboratory shaker (250 rpm, RT) in 45 mL centrifuge tubes filled to 35 mL. The non-modified samples also underwent this procedure to evaluate the necessity of CS-g-PCL modification for the alginate/CS-TGF-β_3_-TPP modification.

Modification with TGF-β_3_-loaded Chitosan-Tripolyphosphate (CS-TPP) nanoparticles was performed according to Roger et al. [34]. To carry this out, TGF-β_3_ (10 µg/mL in citric acid 10 mM, −80 °C) was dissolved in CS42 (42% DA via acetylation of the same chitosan from Sigma-Aldrich, Mn = 190,000–310,000) solution (1 mg/mL, Acetic acid 0.1% (*v*/*v*) in Milli-Q^®^). The resulting solution was mixed with TPP (Milli-Q^®^, 1 mg/mL) in a 3:1 volume ratio regarding CS/TPP, yielding a CS-TGF-β_3_-TPP nanoparticle suspension containing 1 µg/mL TGF-β_3_.

Under a clean bench, sterilized CS-g-PCL_56_/Alginate-scaffolds were immersed into the CS-TGF-β_3_-TPP nanoparticle suspension in Sorenson tubes (2 mL, low protein binding, Sorensen BioScience, Salt Lake City, UT, USA) for 20 min with one scaffold per tube. After removal from the tubes, the scaffolds were laid on low-lint paper towels to remove excess loading suspension. The scaffolds were then moved to tubes containing Milli-Q^®^ for 1 min and another group of such tubes for another minute as a final rinsing step for excess nanoparticle removal. The scaffolds were removed from the tubes, and excess water was removed again using low-lint paper and successive vacuum drying at RT. All experiments were conducted with 4 samples per species, each within a separate tube. However, the electro-spun fiber mats ((8 × 16) mm^2^ cutouts), which serve as a reference benchmark, were incubated in groups of 4 per tube.

### 2.2. Cell Culture

#### 2.2.1. Scaffold Seeding

Human primary mesenchymal stem cells (MSCs) were isolated from the femoral heads of patients undergoing total hip arthroplasty upon written consent and allowance from the local ethics committee (EK 300/13). The MSCs originating from a male donor, 65 years old, were used in cell culture passage four. Scaffolds were seeded at a cell density of 500,000 cells/cm^2^; i.e., SFMU scaffolds (A = 1.5 cm^2^) were seeded with 750,000 cells per scaffold and ROMO (A = 1 cm^2^) scaffolds with 500,000 cells. Cells were resuspended in 200 µL (SFMU scaffolds) and 166.6 µL (ROMO scaffolds) of Glutamax medium (Life Technologies, Carlsbad, CA, USA) to ensure adequate impregnation of the scaffold. The samples were incubated for 1.5 h at 37 °C and 5% CO_2_ to ensure the adhesion of cells to the scaffold and to prevent cells from being rinsed out of the fiber structure. Next, 4 mL of the medium (DMEM GlutaMAX (low glucose), Life Technologies, Carlsbad, CA, USA) supplemented with 10% fetal calf serum (FCS) (FBS Premium Fetal Bovine Serum, PANBiotech GmbH, Aidenbach, Germany), 1% penicillin-streptomycin (Merck KGaA, Darmstadt, Germany), and 1% non-essential amino acids (MEM NEAA, Life Technologies, Carlsbad, CA, USA) was added. The following day, the medium was changed to a maintenance medium with the following composition: DMEM GlutaMAX (high glucose) (Life Technologies) supplemented with 10 FCS (FCS, PANBiotech GmbH), 1% penicillin-streptomycin (Merck KGaA), 25 µg/mL Vitamin C (L-Ascorbic acid 2-phosphate sesquimagnesium salt hydrate, Merck KGaA), and 5 ng/mL b-FGF-4 (Human recombinant FGF-4 (Stemcell Technologies Inc., Vancouver, BC, Canada)). Subsequently, the medium was changed every other day. The MSCs were cultivated for 14 days. The analyses described below were performed on day 14, or the cells were fixated in the scaffold on that day for further analysis.

#### 2.2.2. Live–Dead Staining

The Invitrogen™ LIVE/DEAD™ Cell Imaging Kit (488/570) (R37601, Thermofisher scientific, Waltham, MA, USA) was used for live–dead staining. The fluorescence staining is based on Calcein AM (4 mM in anhydrous DMSO) and BOBO™-3 Iodide (1 mM solution in DMSO). The staining was conducted according to the product’s protocol. For imaging, a fluorescence microscope DMI 4000B (Leica Microsystems GmbH, Wetzlar, Germany) was used.

### 2.3. Analytics

#### 2.3.1. Tensile Tests

Before testing, all samples were stored at standard conditions according to DIN EN ISO 139 [39] for at least 24 h. The yarn count was determined according to DIN EN 13392 [40]. Uniaxial tensile tests for fibers were performed following DIN EN 13895 [41] (monofilaments) and DIN EN ISO 2062 [42] (multifilaments) using an automatic tensile testing device (STATIMAT 4U, Textechno, Herbert Stein GmbH & Co. KG, Mönchengladbach, Germany). The scaffolds were tested with a clamping length of 40 mm for comparability with the native cruciate ligament length. A speed of 40 mm/min was used. All scaffolds were prepared with cardboard force transmission elements. The tests were performed on the universal testing machine ZmartPro from ZwickRoell GmbH & Co. KG, Ulm, Germany.

#### 2.3.2. Scaffold Morphology

The porosity of the three-dimensional scaffolds was determined using µ-CT scans. For evaluation, the application PoroDict of the program GeoDict (GeoDict 2014, Software module: PoroDict, Math2Market GmbH, Kaiserslautern, Germany) was used. The braiding angle and braiding density were determined using light microscopy. Per braided scaffold, three samples were analyzed at three positions. Per position, the braiding angle, as well as the braiding density, was analyzed at three points (n_total_ = 15).

#### 2.3.3. Sterilization

The sterilization of the scaffolds was conducted according to DIN EN ISO 11137-1 [43] by Mediscan (Kremsmünster, Österreich). Electron beam sterilization with an intensity of 25 kGy was used as described in de Cassan et al. [44]. The mechanical properties of the braided, two-layered samples with and without sterilization were analyzed in tensile tests as described in Section 2.3.1.

#### 2.3.4. Confocal Laser Scanning Microscopy (CLSM)

CLSM was carried out on a ZEISS CLSM-510 Meta scan head connected to an Axiovert 200M (Oberkochen, 73447, Germany). Measurement included multiple scans at various depths of the CS-g-PCL_56_/alginate fluorescein amine scaffolds, with 5× and 40× magnification at 488 nm excitation and 528 nm emission wavelength. The gain was set to 959 with a pinhole setting of 8.18 AU/158 µm. In addition, lambda scans were performed to verify that the detected light was a result of fluorescence and not a reflection of any other source. Data were processed with ZEN lite 3.7 (blue edition) by ZEISS (Oberkochen, Germany).

#### 2.3.5. Scanning Electron Microscopy (SEM)

After incubation, the samples were washed twice with PBS before fixation with 3% glutaraldehyde solution for at least 24 h. The SEM analysis was conducted by the Electron Microscopy Facility of University Hospital Aachen, Aachen, Germany. The samples were washed for 15 min with 0.1 M Soerensen’s-Phosphate buffer (Merck, Darmstadt, Germany). Afterward, the samples were incubated stepwise in 30%, 50%, 70%, 90%, and 100% (*v*/*v*) ethanol for 10 min each. The last step was repeated three times. The samples were dried in the air. The dried samples were coated with 10 nm gold-palladium in a sputter coater (Sputter Coater EM SCD500, Leica, Wetzlar, Germany) and investigated with a SE-microscope (ESEM XL 30 FEG, FEI, Philips, Eindhoven, The Netherlands).

#### 2.3.6. 2-Photon Laser Scanning Microscopy

Cell attachment on fiber level as well as cell morphology was visualized by 2-photon laser scanning microscopy (TPLSM; Fluoview FV1000 MPE, Olympus Life Science, Tokyo, Japan) equipped with a 25× water immersion lens (XLPLN 25× NA 1.05, Olympus Life Science, Tokyo, Japan) and a MaiTai Deep-See Titan-Saphir laser (Spectra Physics, Milpitas, CA, USA). Images were taken at random spots. Image processing was performed using the software ImageJ (https://imagej.net/ij/, accessed on 20 May 2021).

#### 2.3.7. Differential Scanning Calorimetry (DSC)

DSC measurements were performed on a DSC 204 thermal analysis device, a CC 200 cooling controller, and a TASC 414/3 A system controller all by NETSCH (95100, Selb, Germany). Samples were analyzed in aluminum crucibles (25/40 μL) with punctured lids by Thepro (52525, Heinsberg, Germany). For gravimetrical measurements, a microbalance type XS3DU by METTLER TOLEDO (35353, Giessen, Germany) was used. All measurements were performed in triplicate with a 10 K/min heating and cooling rate with 3 cycles per sample.

#### 2.3.8. Gel Permeation Chromatography (GPC)

GPC measurements were performed on a PSS SECcurity^2^ instrument (PSS, Mainz, Germany) equipped with a SECcurity^2^ vacuum degasser, a SECcurity^2^ TCC6500 column oven, an Agilent Infinity 1200 isocratic pump, a PSS SECcurity^2^ 1200 refractive index detector (relative measurement against polystyrene standard), and a manual injection valve from Rheodyne. All species were recorded in THF at 40 °C on a PSS triple SDV column setup (1× pre-column, 2× main columns, each 10 µm particle, and pore size) with a concentration and flow rate of 1 g/L and 1 mL/min, respectively.

#### 2.3.9. Release and ELISA of TGF-β_3_

The TGF-β_3_-loaded scaffolds were immersed into Sorenson tubes (2 mL, low protein binding, Sorensen BioScience, Salt Lake City, UT, USA) containing 1 mL PBS with BSA (0.1% (*w*/*v*)) and NaN_3_ (0.02% (*w*/*v*)) added. The release was performed at 37 °C in a climate chamber with the supernatant being exchanged after 1, 8, 25, 96 h, and onwards until day 32. Four samples per species were incubated in separate tubes, and their supernatant was collected and stored at −20 °C for later ELISA analysis. In the case of the electro-spun reference fiber mats, 4 samples were incubated in one tube. For quantification of the released human TGF-β_3_ in the supernatant, an ELISA kit ((DY243, 15 plates) and a duo-set ELISA Ancillary Kit 2 (R&D System, Minneapolis, MN, USA) were used. The calibration was derived from a series of bulk protein solutions. As measured, the absorbance of 3,3′,5,5′-tetramethylbenzinidine at 450 nm was detected using an Infinite^®^ 200 PRO (Tecan Group Ltd., Männedorf, Switzerland).

#### 2.3.10. Statistical Analysis

The analysis, presented in the graphs and tables, is specified in the form of mean ± standard deviation. It was statistically examined using an unpaired two-sample *t*-test. A significance level of 5% was applied. The statistical analysis was carried out using the software Microsoft Excel 2016 (Microsoft Corporation, Redmond, WA, USA).

## 3. Results

### 3.1. Preparation of PCL Fibers

**Figure 1:**
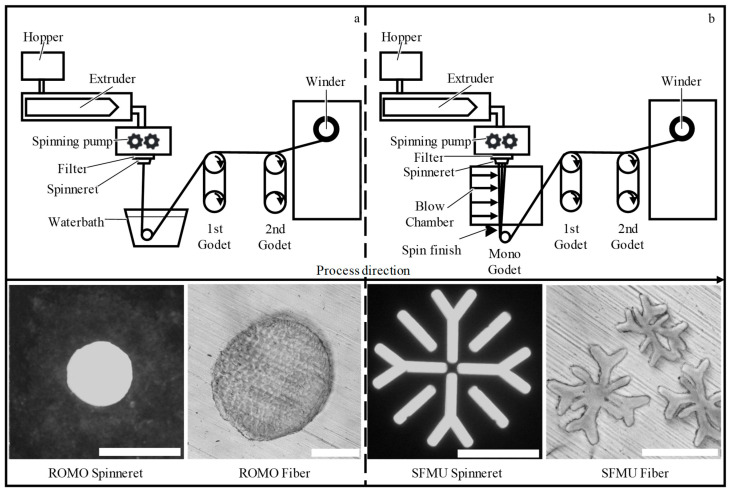
Process schematic of filament production for (**a**) round monofilaments (ROMO) and (**b**) snowflake-shaped multifilaments (SFMU). Optical microscopic images show the spinnerets used for the fabrication of round and snowflake-shaped fibers as well as the resulting fiber cross-sections (based on [26]). Scale bars: 500 µm (spinnerets) and 100 µm (fiber cross-sections).

PCL fibers were fabricated via melt spinning. Using respective spinnerets, **ro**und **mo**nofilaments (ROMO) and **s**now**f**lake-shaped **mu**ltifilaments (SFMU, with 10 filaments per yarn) were produced (Figure 1).

Yarn count (1 dtex = 1 g/10,000 m) and mechanical properties comprising maximum tensile force (F_max_) are displayed in Table 1.

### 3.2. In Vitro Degradation and Molecular Weight Analysis

**Figure 2:**
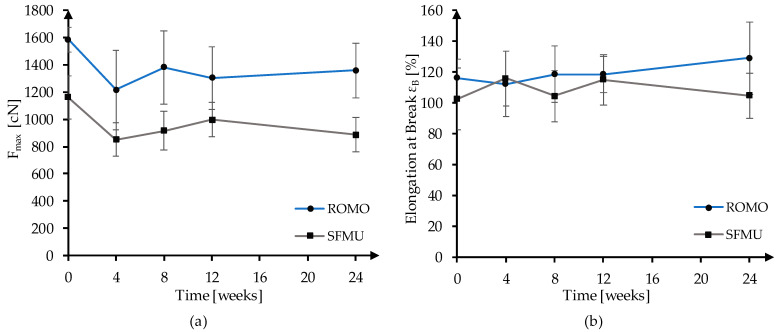
Maximum force (**a**) and elongation at break (**b**) of PCL fibers during in vitro hydrolytic degradation for 24 weeks in PBS at 37 °C.

Long-term mechanical stability was evaluated in an in vitro study for 24 weeks in PBS at 37 °C. After 4, 8, 12, and 24 weeks, 30 samples were extracted and mechanically characterized. The development of the maximum force (F_max_) and elongation at break (ε_B_) is displayed in Figure 2. F_max_ decreased significantly between t_0_ (no degradation) and t_4_ (24 weeks) for both fiber morphologies. ROMO and SFMU lost 224.95 N (*p* ≤ 0.0001) and 274.51 N (*p* ≤ 0.0001) during the 24 weeks of degradation, which corresponds to a strength loss of about 14.20% and 23.65%, respectively. No embrittlement was observed during the degradation study. While ε_B_ did not change significantly for SFMU (*p* = 0.635), an increase (*p* = 0.012) from 116% to 129% was observed for ROMO in the same time frame.

**Figure 3:**
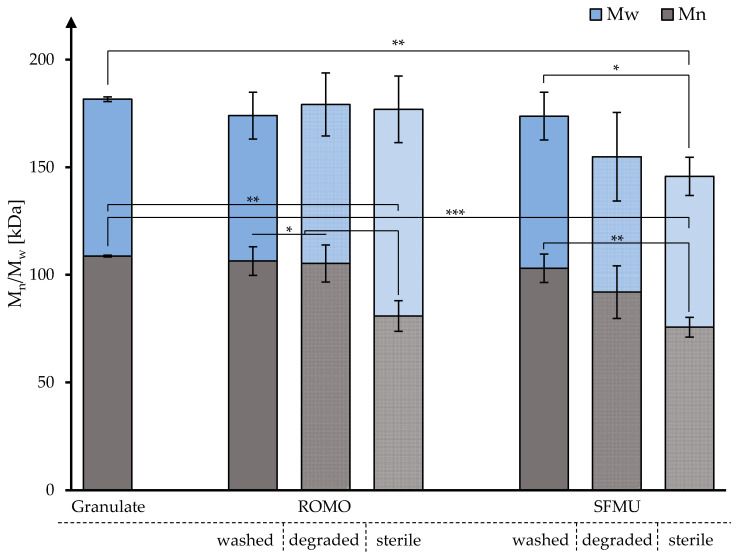
M_n_ and M_w_ across processing stages of ROMO and SFMU fibers obtained via GPC. (* *p* ≤ 0.05; ** *p* ≤ 0.01; *** *p* ≤ 0.001).

The molecular weight distribution of the raw material (granulate, n = 3), fibers from washed scaffolds, in vitro degraded fibers, and fibers from non-degraded sterilized scaffolds (each n = 1) was obtained via GPC to obtain further information on the impact of fabrication, degradation, and sterilization on the observed decline in mechanical strength (Figure 3).

For both ROMO and SFMU species, no significant decline in M_n_ and M_w_ was observed for the washed scaffolds compared to the raw granulate. This was also applicable to the degraded ROMO and SFMU scaffolds. Electron beam sterilization, however, lowered M_n_ for both species significantly when compared to granulate and their non-degraded counterparts. M_w,_ however, was only lowered significantly compared to granulate and non-degraded samples with the SFMU species, whilst the ROMO’s M_w_ did not significantly change. Detailed data are given in Table 2.

**Figure 4:**
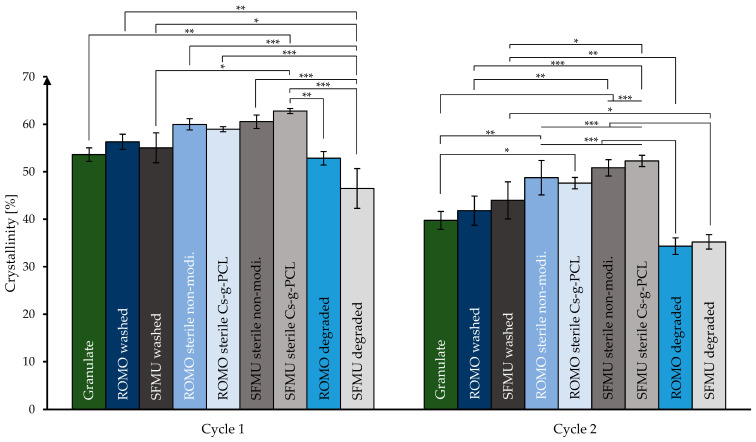
Crystallinity during processing stages of ROMO and SFMU scaffolds obtained via DSC. (* *p* ≤ 0.05; ** *p* ≤ 0.01; *** *p* ≤ 0.001).

De Cassan et al. [44] showed that for PCL-based electro-spun fibers, the fabrication, CS-g-PCL modification, and sterilization via β, γ, and X-rays have a huge impact on various material properties, in particular the degree of crystallinity of the fibers. As a change in scaffold crystallinity not only influences the mechanical properties but could also have an impact on the self-induced crystallization of CS-g-PCL on the PCL scaffold, all scaffolds and the granulate were investigated via DSC (Figure 4).

Especially the first loop of the measurement is of interest as it emphasizes the influence of the processing step on the crystallinity. A two-sample *t*-test was performed in order to evaluate the significance of apparent changes in crystallinity with a significance level of at least * ≤ 5%. All further results referred to as significant satisfy this criterion. Both washed scaffold species did not show a significant increase in crystallinity compared to the granulate. However, after electron beam sterilization, there was a significant increase in crystallinity compared to the non-treated scaffold species of both ROMO and SFMU. This increase of 4.9% on average across both species is in line with the findings of de Cassan et al. for similarly treated samples [44]. The modification with CS-g-PCL_56_, however, only showed a significant increase in crystallinity in the case of the SFMU species. This could be due to the larger surface area of SFMU scaffolds exposed to radiation compared to the ROMO species as well as due to the modification itself. The degraded samples showed in both cases a significant decrease in crystallinity compared to their washed counterparts. This is especially pronounced for SFMU species, which might be again due to their higher specific surface area compared to ROMO-type scaffolds. In the second loop, the relations stay the same although the overall crystallinity is significantly lowered. Comparing the second and third loops indicates that there is no more thermal history from the processing present (see Supplementary Materials).

### 3.3. Scaffold Characterization

**Figure 5:**
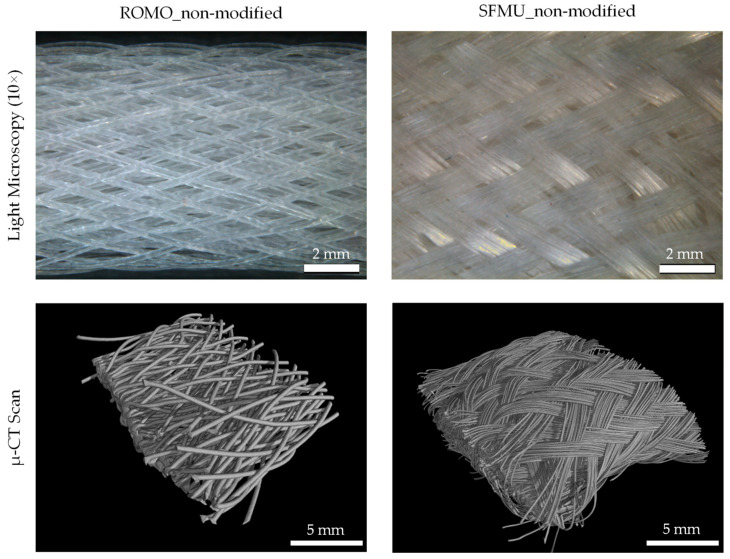
Light microscopy and µ-CT scans of non-modified 4-layer (192 filaments) braids with ROMO and SFMU fibers.

The morphological properties of the three-dimensional scaffolds were investigated by µ-CT and light microscopy. The surface of the scaffolds and the three-dimensional view are depicted in Figure 5. The different filament structure is also shown in the deposition in the scaffolds.

The SFMU filaments form a denser braid due to the side-by-side deposition of the single filaments. The porosity of the SFMU braids indicates a decrease of about 14% by changing from two to four braid layers. The ROMO braids show a reduction of only about 2.5%, from 86.31 ± 0.02% for the two-layer braids to 83.73% for the four-layer braids. The braid angle is between 19.61° ± 2.07° (ROMO two-layer) and 24.19° ± 1.52° (SFMU four-layer). The braid angle of the two-layer braids is slightly lower for both fiber morphologies. Since the same production settings were used for the layers, this is explained by the formation of a core with increasing diameter through the layers. The cross-section of the samples has an elliptical shape. The width of the specimens limits their implantability with present surgical methods, since these use a 9 mm diameter of the bone channel. The width of the specimens is 6.58 mm ± 0.66 mm (ROMO) and 8.86 mm ± 0.79 mm (SFMU) for the four-layer braids. Thus, implantability is not impeded. The morphological characteristics of all scaffolds are shown in Table 3.

**Figure 6:**
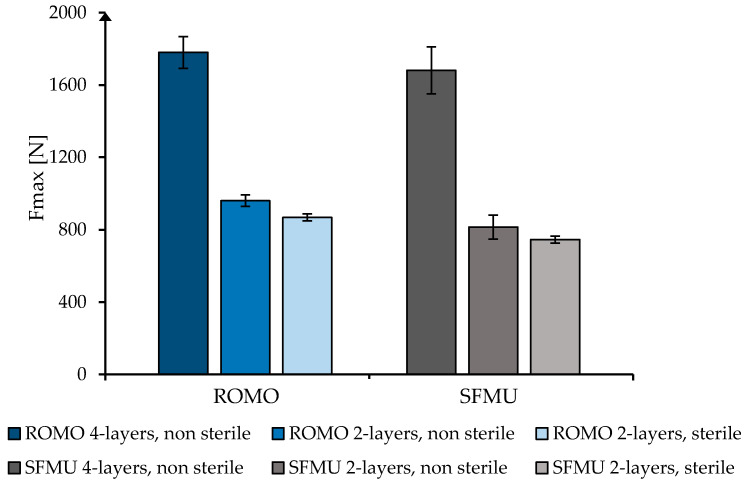
Maximal tensile load of the braided scaffolds based on the filament type, number of layers and sterilization.

Figure 6 shows the mean value with standard deviation of the maximum tensile load (F_max_) of the braids in the uniaxial tensile test. The highest mean value was determined for ROMO four-layer at 1780.2 ± 87.8 N. The SFMU four-layer braids show a maximum tensile load of 1681 ± 129.3 N. Thus, the maximum tensile force is comparable to that of a native human cruciate ligament (1730–2160 N) [45,46].

The braids of both filament types show good scalability, as the two-layer braids reach about 49% of the maximum tensile load compared to the four-layer braids (ROMO two-layer 960.1 ± 31.4 N, SFMU two-layer 814.9 ± 65.7 N). The effect of sterilization on the mechanical properties was investigated using the two-layer braids. F_max_ was reduced by about 9.6% to 867.8 ± 19 N for ROMO 2-layer_sterile and by about 8.5% to 745.7 ± 19.4 N for SFMU 2-layer_sterile.

### 3.4. Live–Dead Assay

**Figure 7:**
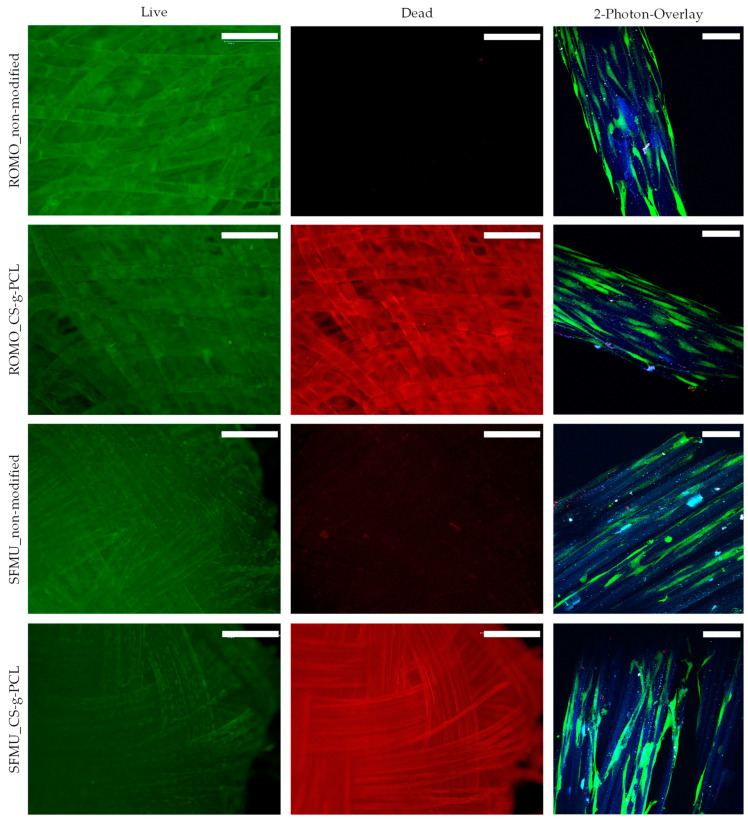
Fluorescence images of the live–dead staining (scale bar = 1 mm) and 2-photon laser scanning microscopy (scale bar = 100 µm) images of the modified and non-modified braided scaffolds with ROMO and SFMU fibers.

Fluorescence microscopic images as well as an overlay of the two-photon laser scanning microscopy images are shown in Figure 7 for ROMO and SFMU four-layer braids with and without Chitosan-graft-PCL surface modification. In this stage, only qualitative observations could be made. Generally, all samples showed high viability with an even distribution and no visible clusters as well as only very few dead cells. The surface modification clearly exhibited auto-fluorescence with the dead staining. Still, dead cells were distinguishable as lighter red dots. Qualitatively, neither the fiber morphology nor the presence or absence of the surface modification appears to have an obvious effect on the number of cells. The two-photon images did not provide information on the dead staining. A light blue auto-fluorescence of the fiber itself was visible, and occasional light bluish-green artifacts were observed. The living cells showed a spindle-shaped morphology and were mainly oriented along the fiber axis, indicating cellular attachment along the fiber axis. In the case of the SFMU braids, a tendency toward growth within the grooves was observed.

**Figure 8:**
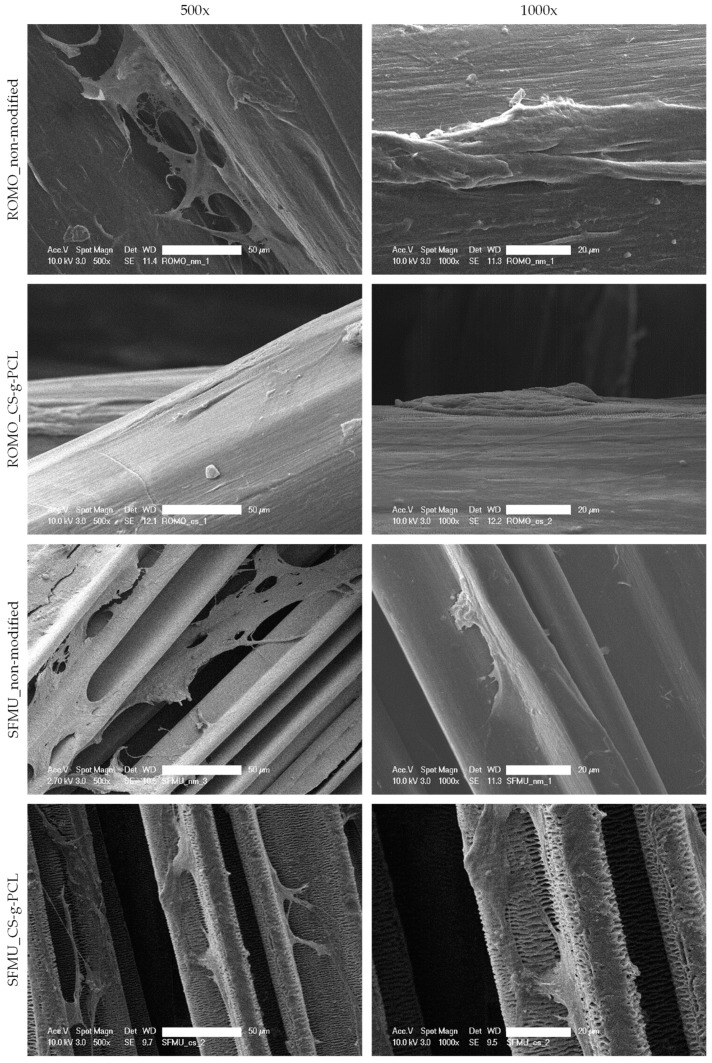
SEM images with magnification of 500× (scale bar = 50 µm) and 1000× (scale bar = 20 µm) of the modified and non-modified braided scaffolds with ROMO and SFMU fibers.

SEM images of the different braided scaffolds with and without Chitosan-graft-PCL surface modification are depicted in Figure 8. The MSCs attached flatly on the surfaces of the scaffold or in the corners that resulted from the grooved surface of the SFMU fibers. Cell protrusions were observed bridging distances that occurred between two fibers or across grooves in the case of the snowflake morphology. While the non-modified samples were smooth, the Chitosan-graft-PCL modified fibers showed a change in surface morphology similar to the shish-kebab surface crystallization observed by de Cassan et al. [32]. The change was particularly pronounced for SFMU.

### 3.5. Surface Functionalization and Release of TGF-Beta

The well-established modification of PCL-based electro-spun fiber mats via induced crystallization of CS-g-PCL on the surface by de Cassan et al. [32] also worked with the yarn-based, high-strength PCL scaffolds. Compared to the electro-spun fiber mats, the yarn-based scaffolds were even more stable in the coating process and more tolerant when modification parameters like the concentration of AcOH in the modification solution or its temperature were altered.

**Figure 9:**
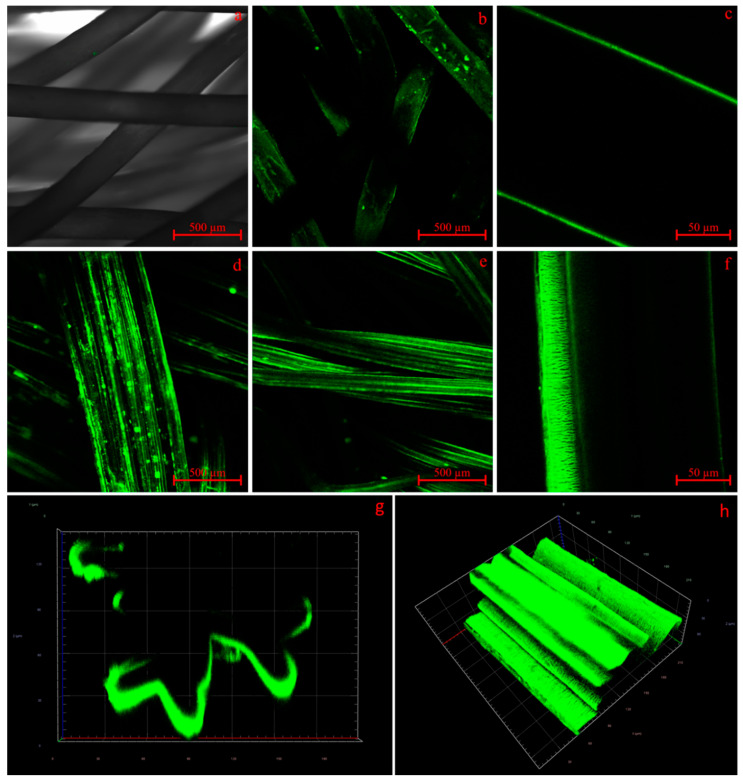
CLSM images of alginate fluorescein amine-treated ROMO and SFMU scaffolds. (**a**) Non-CS-g-PCL-modified ROMO including transmitting light channel, (**b**,**c**) CS-g-PCL_56_-treated ROMO, (**d**) CS-g-PCL_56_-treated SFMU before supersonic treatment, (**e**,**f**) CS-g-PCL_56_-treated SFMU after supersonic treatment, (**g**) cross-section of a CS-g-PCL_56_-treated SFMU from 3D scan, and (**h**) 3D scan of CS-g-PCL_56_-treated SFMU.

For clarification, if the observed surface change in SEM was due to the presence of CS-g-PCL on the scaffolds, alginate fluorescein amine was applied to the surface in a dip coating procedure, and the surfaces were subsequently analyzed by CLSM. Alginate species are known only to attach to the surface of PCL if its surface charge is positive due to the CS-g-PCL as the surface charge of bare PCL tends to be negative. CLSM showed that modification with CS-g-PCL was successful (Figure 9). The non-CS-g-PCL-modified samples (Figure 9a) did show only very little fluorescence. However, the intensity was so low it was necessary to superimpose the transmitted light channel on the fluorescence channel to make the ROMO scaffold structure visible as the fluorescence signal alone was far too weak and inhomogeneous to display the structures. The CS-g-PCL-modified ROMO and SFMU scaffolds b–h, on the other hand, showed strong uniform fluorescence. In the focal plane of the CLSM, the intensity was high enough to obtain clean images of both species. On both species, agglomerates of the Alg-Fa (Alginate Fluorescein amine) were visible although the dipping solution was filtered before modification. Hence, the agglomerates should have formed after the modification process. A 2 min submersion in an ultrasonic bath (Milli-Q^®^) at RT reduced the number of agglomerates significantly (e vs. d) compared to samples without that treatment, whilst the intensity and homogeneity remained at a similar level. On the SFMU species f and h, it was possible to identify the change in surface morphology earlier discussed in Section 3.4. In addition, the cross-section image and 3D scan of g–h showed that the modification with Alg-Fa is only present on the surface. This is in line with the findings of de Cassan et al. [32]. As the modifiability with Alg-Fa could be regarded as an indirect measure for Alg modifiability in general, the homogeneity and intensity of its fluorescence should be linked to the amount of Alg applicable to the implant surface. This in turn should directly affect the maximum possible amount of CS-TGF-β_3_-TPP present on the fully modified and loaded implant scaffolds. A later modification was implemented similar to the dip coating application of Alg(-Fa). Therefore, TGF-β_3_ was added in situ to the ionic gelation process of CS and TPP, forming suspended nanoparticles. The CS-g-PCL_56_/ALG-modified scaffolds were then dip-coated using the resulting suspension, yielding CS-g-PCL_56_/ALG/CS-TGF-β_3_-TPP modified scaffolds for later release testing.

**Figure 10:**
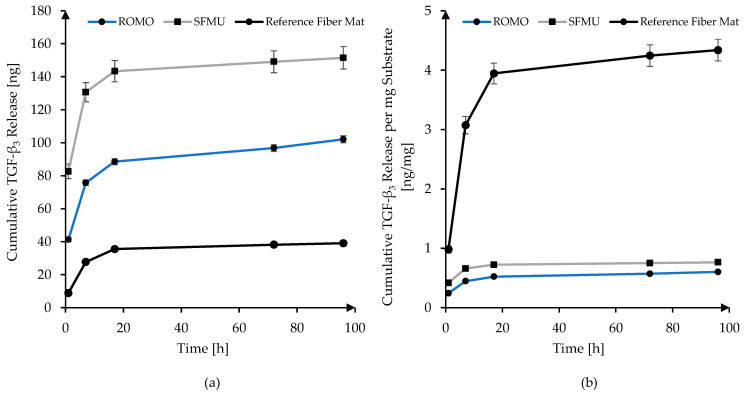
Cumulative in vitro release of TGF-β_3_: (**a**) absolute; (**b**) per milligram of substrate.

The release of TGF-β_3_ from the CS-g-PCL/alginate/CS-TGF-β_3_-TPP modified scaffolds was successfully monitored with an ELISA over 32 days and showed promising results (Figure 10a) compared to electro-spun PCL fiber mats, which were also monitored for referencing. The direct release from CS-TGF-β_3_-TPP nanoparticles and fS-g-PCL/alginate/CS-TGF-β_3_-TPP modified fiber mats is a well-established procedure according to studies by Roger et al. [34], Sundermann et al. [47], and Berten-Schunk et al. [48]. Although the release kinetics are not yet optimal and show a strong burst release in the first 24 h, the amount of TGF-β_3_ released from the yarn-based scaffolds was quite promising as it should suffice at inducing cell differentiation specific to the growth factors family according to Roger et al. [34], Berten-Schunk et al. [48], Barry et al. [49], and Mueller et al. [50]. Electro-spun fiber mats showed a comparably low amount of growth factors released, but this is due to their size. The electrospinning process, for technical reasons, can only produce electro-spun fiber mats with a limited thickness, whereas yarn-based scaffolds are theoretically unlimited in all three dimensions. The higher specific surface area of the electro-spun fiber mats compared to the yarn-based scaffolds apparently cannot make up for this disadvantage. With respect to the amount released per milligram of substrate, the electro-spun fiber mats perform between 5.7 and 7.2 times better than the yarn-based scaffolds due to their high specific surface area (Figure 10b). However, this advantage becomes irrelevant due to their absolute limitations in thickness. The SFMU species releases about 27% more TGF-β_3_ per milligram of substrate compared to the ROMO species. Surprisingly, the relative amounts do not scale linearly with the specific surface areas calculated for both species.

## 4. Discussion

Tendon and ligament scaffolds have to fulfill numerous requirements, which include mechanical, degradation, processual and biological aspects. Textile technology gives access to the fabrication of 3D macroporous scaffolds with adjustable mechanical properties and morphology, but many of these requirements are directed toward the material itself. In our earlier works, PCL fibers were found promising in terms of mechanical properties, strength retention during PBS exposure, and processability with circular and hexagonal braiding [26,51]. However, biological aspects such as the general cell response and the possibility to provide biological cues have not yet been investigated. In this study, a versatile material system based on sufficiently slow-degrading melt-spun fibers, scaled up to human ACL while at the same time offering biological cues, was introduced and biologically examined for the first time, indicating generally high cell viability. Two pathways were pursued to provide potential biological stimuli. On the one hand, for the first time, melt-spun PCL was successfully modified with a Chitosan-graft-PCL surface modification. On the other hand, also for the first time in the context of ACL scaffolds, the influence of non-circular PCL fiber cross-sections on cell morphology was investigated. In addition, surface functionalization linked to the CS-g-PCL enabled the release of the growth factor TGF-β_3_. The release amount of TGF-β_3_ was—as a surface-dependent effect—adjustable through the fiber cross-section. The effect of the sterilization method on the mechanical properties of the scaffolds was also investigated, showing a reduction of ~9% of the scaffolds F_max_.

Mechanically scaled-up, degradable scaffolds for human ACL have been investigated as different textile structures and consisting of different fiber materials. For example, Hahn et al. fabricated embroidered scaffolds from PLA/PLCL fibers [10,52]. Mengsteab et al. investigated 3D-braided PLLA scaffolds and showed scalability to the mechanical properties of a human ACL [53]. Cooper et al. used PLAGA fiber material in a braided scaffold [54]. Laurent et al. investigated multilayer-braided PLCL scaffolds [55]. The group of Freeman, Laurencin et al. thoroughly examined braided, twisted, and braid-twisted scaffold designs from PLLA and PLAGA [18,19,56]. Despite their large potential, melt-spun PCL fibers have scarcely been investigated in the context of ACL scaffolds. Leroux et al. have investigated melt-spun PCL fibers and knotted fiber bundles [27,28]. Recently, the melt-spun PCL was further processed into knitted scaffolds with a focus on osseointegration [29]. The knitted scaffolds revealed the high potential of especially poly (sodium styrene sulfonate) grafted PCL in vitro as well as in vivo, but no mechanical characterization was reported. Investigations on scaffolds that are scaled up to meet the primary stability necessary for human ACL (male: 1119–2517 N, female: 739–1793 N [2,57]) have not been performed with PCL to date. In the present study, circular braided scaffolds consisting of 4 × 48 filaments were produced from circular monofilaments as well as snowflake-shaped multifilaments, thus providing a broad range of specific surface area. Even though the scaffolds were not optimized in terms of tensile strength (compared to our other works), they exhibited acceptable primary stability of 1780 N and 1680 N, respectively. The multilayer braided scaffolds are macroporous structures with porosities of 73% and 83%, respectively. Hollister et al. stated that a porosity of 60–70% was beneficial for cellular ingrowth [58]. Simultaneously, the pore size should be higher than 100–250 µm to ensure sufficient vascularization and tissue ingrowth [17,59]. The filament type strongly affects the pore size distribution of the scaffold, with the multifilament scaffold exhibiting smaller pores than the monofilament scaffold. In addition to the filament type, the morphological properties such as pore size and porosity can also be adjusted by the braiding parameters braiding angle and braiding pattern [55,60]. These also have a major influence on the mechanical properties such as elongation, ultimate tensile strength, and stiffness of the braids [51,54,55]. This allows the scaffolds to be adapted to the mechanical properties of the human ACL.

For both fiber morphologies, a Chitosan-graft-PCL surface modification was applied (its presence visualized using CLSM) and tested against the unmodified reference. The modification has previously been investigated in the context of electro-spun PCL fiber mats exhibiting increased initial cell attachment in the first 24 h after seeding [32]. However, the non-modified fiber mats almost caught up after 72 h. The yarn-based samples showed generally high cell viability. An obvious effect of either the fiber morphology or the surface modification on the cell viability was not observed. However, due to the three-dimensionality of the scaffold, we refrained from a quantitative analysis of the cell viability in this stage, which will be amongst the next steps. The surface-modified samples exhibited obvious auto-fluorescence with the dead staining [61]. A major advantage of the Chitosan-graft-PCL surface modification is the possibility to attach functionalization agents such as drugs or growth factors. We showed the feasibility of attaching and releasing TGF-β_3_ for both fiber morphologies (ROMO and SFMU). The amount of released TGF-β_3_ within 96 h was 102 ± 2.1 and 151 ± 6.75 ng, respectively. In the scope of Roger et al. [34] and Berten-Schunk et al. [48], a concentration of 10 ng/mL was identified in in vitro studies to be effective, and, therefore, both species should be capable of delivering the necessary amounts of growth factors. However, the burst release could pose a problem when it comes to both in vitro and in vivo studies. In this regard, an approach with blocking layers could help moderate the release and prolong the window for improved cell differentiation [62]. The release amount was significantly higher for the SFMU scaffolds compared with the ROMO scaffolds, indicating that it can be tailored to some extent by adjusting the specific surface area of the fiber. However, the release amount did not increase proportionally with the specific surface area. A more detailed examination of the interplay of fiber morphology and release kinetics has to be conducted in future trials.

Independent of release agents, the use of non-circular fibers to promote biological properties by providing increased surface area for cellular attachment as well as potential growth guidance mediated by the grooves has been investigated before [35,36]. While no obvious effect of the fiber morphology on the cell viability was observed in the present study, a tendency of growth within the grooves and along the fiber axis was perceived. Whether cell adhesion and proliferation can be promoted through a modified fiber cross-section geometry is likely subject to a more complex interplay of different influence factors such as cell type, cultivation conditions, and surface topography, making further investigations necessary [63]. The study makes further biological investigation necessary to quantify the potential effects of the fiber morphology as well as the surface modification. Besides that, future studies will address the effect of growth factor release on the cell response.

## 5. Conclusions

Tissue engineering of tendons and ligaments is considered a promising approach to overcome the deficiencies of current procedures. However, the requirements for a suitable scaffold are very comprehensive and range from mechanical, degradation-related, and scalability-related aspects to biological aspects. Macroporous textile scaffolds made from adequate fiber material have the inherent potential to meet the first three of these requirements. In this study, starting from a fiber with suitable strength and degradation behavior scaled to a macroporous scaffold with the dimensions of a human anterior cruciate ligament, we aimed to investigate its biocompatibility as well as functionalizability. In terms of functionalization, two paths were followed. On the one hand, two different cross-sectional geometries with substantially different specific surface areas were used at the fiber level. On the other hand, a CS-g-PCL surface modification was applied onto melt-spun substrates for the first time. All scaffolds exhibited positive cell response. With the CS-g-PCL modification, a platform to incorporate functionalization agents such as growth factors was created. The fiber geometry was identified to be a manipulating variable for the release quantity. Taken together, scaled, surface-modified textile scaffolds present a versatile technology with which the complex requirements of tissue engineering of ligaments and tendons, as well as other structures, can be successfully addressed.

## Figures and Tables

**Table 1:** Properties of the melt-spun PCL fibers.

Fiber	Cross-Section	Yarn Structure	Draw Ratio	Yarn Count [dtex]	F_max_ [cN]
ROMO	Round	Monofilament	5.58	458.6 ± 5.1	1644 ± 100
SFMU	Snowflake	Multifilament	5.58	436.7 ± 7.5	1274 ± 168

**Table 2:** The molecular weight of PCL granulate, melt-spun fibers, degraded fibers, and fibers from sterilized scaffolds. Measurement uncertainty (in %) is given in brackets.

	Granulate		Melt-Spun Fiber		Degraded Fiber (after 24 Weeks)		Sterilized Sample	
Fiber	M_n_ t4 [kDa]	M_w_ t4 [kDa]	M_n_ t4 [kDa]	M_w_ t4 [kDa]	M_n_ t4 [kDa]	M_w_ t4 [kDa]	M_n_ t4 [kDa]	M_w_ t4 [kDa]
ROMO	109 (0.35)	184 (0.16)	106 (6.24)	174 (6.24)	105 (8.18)	179 (8.18)	81 (8.75)	177 (8.74)
SFMU			103 (6.4)	174 (6.39)	92 (13.28)	155 (13.27)	76 (6.07)	146 (6.07)

**Table 3:** Morphological characteristics of the braided scaffolds.

Scaffold	Pix/cm	Braiding Angle α [°]	Porosity P [%]	Width [mm]
ROMO_4-layer	3.84 ± 0.37	21.12 ± 0.97	83.73 ± 0.00	6.58 ± 0.66
ROMO_2-layer_unst	4.50 ± 0.51	19.61 ± 2.07	86.31 ± 0.02	6.19 ± 0.10
ROMO_2-layer_st				
SFMU_4-layer	4.53 ± 0.50	24.19 ± 1.52	73.70 ± 0.00	8.86 ± 0.79
SFMU_2-layer_unst	4.52 ± 0.44	21.37 ± 2.55	87.00 ± 0.05	6.35 ± 0.83
SFMU_2-layer_st				

## Data Availability

Data are contained within the article.

## Supplementary Materials

The following supporting information can be downloaded at: https://www.mdpi.com/article/10.3390/polym16040488/s1. Figure S1: Crystallinity during processing stages of ROMO and SFMU scaffolds obtained via DSC in cycles 2 and 3.
