# Supplementary material for: Topographically and Chemically Enhanced Textile Polycaprolactone Scaffolds for Tendon and Ligament Tissue Engineering
Source: Polymers (Basel). 2024 Feb 9;16(4):488. doi: 10.3390/polym16040488 (PMC10893359; doi:10.3390/polym16040488)
Supplement: Supplementary file 1 [file polymers-16-00488-s001.zip › polymers-2824589-supplementary.pdf]

Supplementary Materials

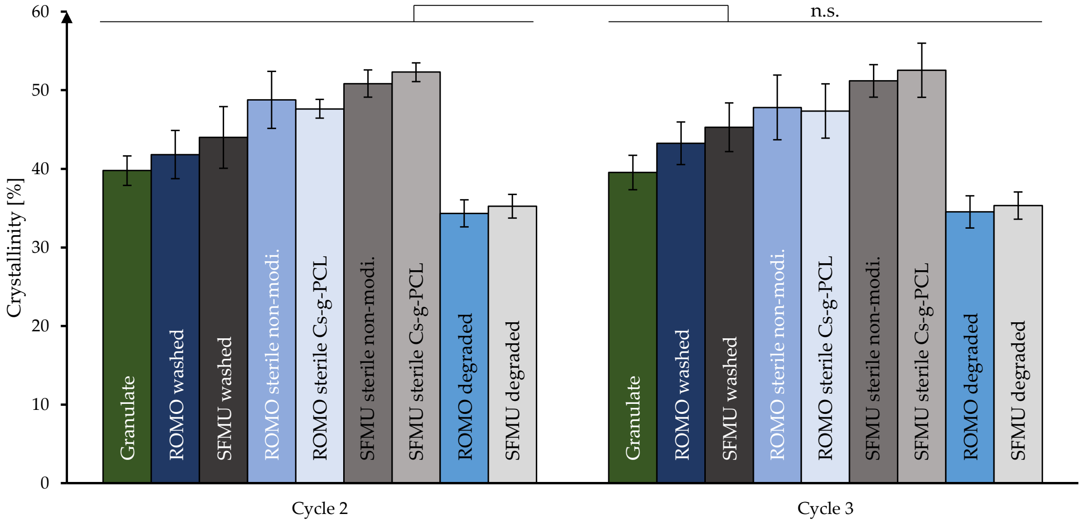

Figure S1. Crystallinity during processing stages of ROMO and SFMU scaffolds obtained via DSC.
